# Epidemiology and outcome of HIV patients in Finland co-infected with tuberculosis 1998–2015

**DOI:** 10.1186/s12879-019-3890-x

**Published:** 2019-03-18

**Authors:** Ville Holmberg, Hanna Soini, Pia Kivelä, Jukka Ollgren, Matti Ristola

**Affiliations:** 1 Clinic of Infectious Diseases, Inflammation Center, Helsinki University Hospital, Post Box 348, 00029 HUS Helsinki, Finland; 2 Department of Internal Medicine, Clinicum, Medical Faculty, University of Helsinki, Helsinki, Finland; 3 Department of Health Security, National Institute of Health and Welfare, Helsinki, Finland

**Keywords:** HIV, Tuberculosis, Outcome, Epidemiology, Co-infection

## Abstract

**Background:**

Tuberculosis (TB) is a major cause of death in HIV patients worldwide. Here we describe the epidemiology and outcome of HIV-TB co-infections in a high-income country with low TB incidence and integrated HIV and TB therapy according to European guidelines.

**Methods:**

This study was based on the HIV cohort of the Helsinki University Hospital which includes all HIV patients in the Helsinki region with a population of 1.5 million. Totally, 1939 HIV-positives who have been under follow-up between 1998 and 2015 were included.

**Results:**

TB was diagnosed in 53 (2.7%) of the HIV-patients. The TB incidence rate was higher in injecting drug users (IRR 3.15; 95% CI 1.33–7.52) and heterosexuals (IRR 3.46; 95% CI 1.64–7.29) compared to men having sex with men. The incidence rate was also higher in those born in Sub-Saharan Africa (IRR 3.53; 95% CI 1.78–7.03) compared to those born in Finland. There was a significant reduction in the total TB incidence rate of 59% per 6-year period between 1998 and 2015 (*p* < 0.001). In injecting drug users there was a reduction in incidence rate from 1182 to 88 per 100,000 (*p* < 0.001) and in people born in Sub-Saharan Africa from 2017 to 195 per 100,000 (*p* < 0.001). Among the 53 HIV-TB co-infected cases, one female and 15 males died during follow up. HIV was the primary cause of death in five patients but none of the deaths were caused by TB.

**Conclusion:**

The incidence rate of tuberculosis among HIV-positives in Finland has been declining between 1998 and 2015. Among injecting drug users, the reduction is probably explained by harm reduction interventions and care in comprehensive care centers in Helsinki. The increased coverage of antiretroviral therapy is probably another main reason for the decline in TB incidence rates. Despite good treatment results for both HIV and TB, the all-cause mortality among Finnish males with HIV-TB was high, and common causes of death were intoxications and suicides.

## Background

Tuberculosis (TB) is the most common opportunistic infection in HIV-positive patients. HIV-TB co-infection was estimated to affect 1.2 million people worldwide in 2015, and to cause 400,000 deaths [1]. HIV-positives are 20–40 times more likely to develop active TB infection compared to HIV-negatives [2]. The most important risk factors for developing active tuberculosis among HIV-positives are low CD4 count, living in high TB-incidence regions and absence of antiretroviral therapy [3–5].

High mortality has been reported in HIV-TB patients in Africa, particularly in patients with advanced disease [6]. In Africa, Asia and Eastern Europe the leading cause of death has been TB. Data about the outcome of HIV-TB co-infections in high-income countries has been limited. A multi-centre study showed that among patients from Western Europe and Argentina, 50% of the deaths during the first three months after the TB diagnosis were TB-related [7]. For patients who died at a later stage the cause of death was predominantly non-TB-related or unknown.

A Cochrane review of 12 randomized treatment trials of latent TB infection in HIV-positives showed a 32% reduction of the incidence of active TB [8]. The reduction was 62% in those with positive tuberculin skin test (TST). Overall, there was no evidence that TB preventive therapy versus placebo reduced all-cause mortality. The need for treatment of latent tuberculosis infection (LTBI) in high-income countries has remained controversial. The WHO [9] and US [10] guidelines recommend testing of all HIV-positives for LTBI and treatment of all patients with positive result in interferon gamma release assays (IGRA) or tuberculin skin test. EACS [11] and BHIVA guidelines [12] recommend IGRA-testing and medication only after risk assessment based on CD4 count, country of origin and length of previous antiretroviral therapy. Despite recommendations for treatment of LTBI, the implementation of this strategy has been limited in high-income countries [4].

The aim of this study was to describe in detail the incidence of TB among HIV-positives and the outcomes of HIV-TB co-infections in a high-income country with low tuberculosis prevalence.

## Methods

### Study participants

This is a retrospective register study based on the cohort of HIV-patients registered for follow up at the Infectious Disease Clinic of the Helsinki University Hospital. This is the only HIV clinic in the Helsinki region in Southern Finland with a population of 1.5 million people, where 63% of all HIV patients in Finland have been diagnosed. The treatment of HIV and tuberculosis is integrated and free of charge for patients living in Finland.

All 1939 patients in the Helsinki HIV Cohort between 1st January 1998 and 31st December 2015 were included in this study. Classified as HIV-TB co-infections were 53 patients diagnosed with an active TB infection in Finland between 1995 and 2015. We excluded four patients, whose tuberculosis diagnosis were made abroad before migrating to Finland.

### Data sources

The InfCare HIV database (RealQ platform, Health Solutions, Sweden) of the Helsinki HIV Cohort has provided the basic data about the patients and details about the TB diagnosis and outcome have been collected from the electronic patient records (Uranus, CGI, Canada) and laboratory database of the Helsinki University Hospital. The categorical variables obtained from the databases were: sex, mode of HIV transmission, country of birth, outcome of TB treatment (WHO classification), MDR TB diagnosed, MAC co-infection diagnosed, AIDS defining diagnoses. The continuous variables obtained were: date of birth, date of death, date of TB diagnosis, date of HIV diagnosis, dates of TB treatment, date of initiation of HIV treatment, CD4 at time of TB diagnosis, CD4 nadir. All new HIV and TB infections in Finland are reported to the National Infectious Disease Register, and from there we have confirmed that all co-infections in the Helsinki region are included in this study. The causes of death were obtained from Statistics Finland.

### Genotyping of *Mycobacterium tuberculosis* isolates

Genotyping was performed with the spoligotyping technique as previously described [13]. The spoligotypes were assigned to international SIT types available in the SITVITWEB database [14] [15]. For spoligotypes for which a SIT code was not available, a local F (Finnish) code was given.

### Statistics

For the statistical calculations, the SPSS Statistics software version 20 (IBM Corporation, NY, USA) and Stata 15.1 (StataCorp LLC, TX, USA) were used. Incidence rates and incident rate ratios and their 95% confidence intervals were calculated using Mantel-Haenszel methods. Score test was used to estimate trends of rates of change in incidence between the different 5-year periods. In the survival analyses of HIV-TB cases Log-rank test was used to calculate *p*-values for categorical variables and Wald test (Cox model) for continuous variables. Hazard ratios and their 95% confidence intervals were calculated using Cox model. Kaplan-Meier plots were used to show estimations of the probability of survival in different subgroups of patients with HIV-TB co-infection.

## Results

### Epidemiology

**Fig. 1:**
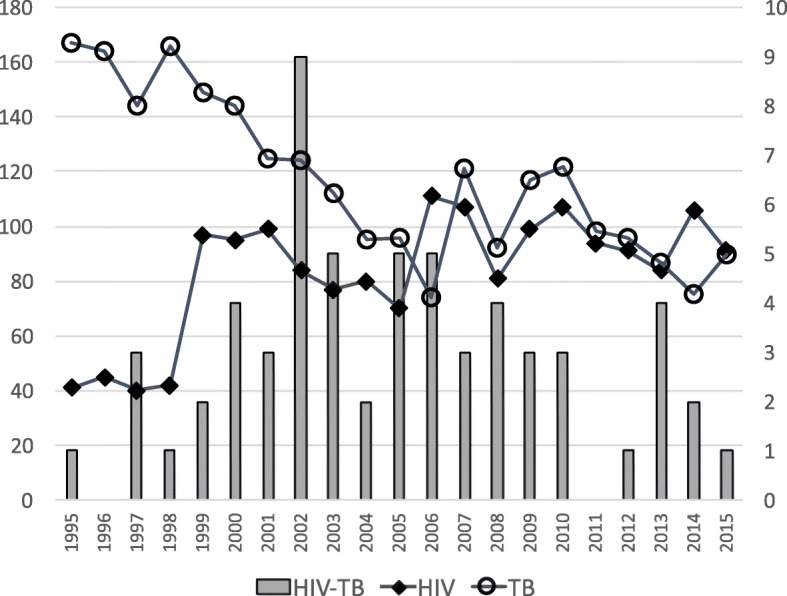
Trends in HIV and TB incidences in Southern Finland. Annual numbers of HIV and TB cases in the Hospital District of Helsinki and Uusimaa in Southern Finland between 1995 and 2015 (left Y-axis). The number of HIV-TB co-infections in the same district are shown by the bars (right Y-axis)

In the patients of the Helsinki HIV cohort there were 53 active TB infections diagnosed in Finland between 1995 and 2015 (Fig. 1). The annual number of new HIV-TB co-infections have mainly been between 1 and 5 in the Hospital District of Helsinki and Uusimaa (HUS) with a population of 1.5 million inhabitants. The annual number of new HIV-infections in the same district has remained stable at a level of about 100 (Fig. 1). The new TB cases have slowly been decreasing from 150 to 100 annually (Fig. 1).

### Baseline data

Between 1998 and 2015 there were 1939 HIV-positives on regular follow up in our cohort (Table 1). Among the HIV-TB cases, the place of birth was Finland in 25 cases and Sub-Saharan Africa in 12 cases. The countries of birth seen in those classified as others were Thailand in 9 cases, Estonia in two cases, Germany in one case, Lithuania in one case and Egypt in one case. The mean CD4 counts at the time of TB diagnosis for the different subgroups presented in Table 1 were the following: males 196 cells per cubic millimetre (range 5–854), females 241 (9–859), IDU 183 (5–634), MSM 303 (10–854), heterosex 194 (9–859), place of birth Finland 221 (5–854), place of birth Sub-Saharan Africa 220 (19–541), place of birth other 146 (9–561).

**Table 1:** Baseline data of the HIV cohort and TB incidence rates

	HIV-Cohort *n* (%)	TB events *n* (%)	Person years^a^	Incidence rate (95% CI)	Incidence rate ratio
Total	1939	53	0.1708	310.3 (237.1–406.2)	
Sex					
Female	534 (27.5)	18 (34.0)	0.0471	382.3 (240.8–606.7)	1.35 (0.77–2.39)
Male	1405 (72.5)	35 (66.0)	0.1237	282.9 (203.1–394.1)	1
Mode of transmission					
IDU	107 (6.4)	12 (24.0)	0.0671	407.5 (231.4–717.6)	3.15 (1.33–7.52)
MSM	755 (45.1)	9 (18.0)	0.0697	129.1 (67.2–248.0)	1
Heterosex	811 (48.5)	30 (60.0)	0.0294	446.9 (312.5–639.3)	3.46 (1.64–7.29)
Place of birth					
Finland	1320 (68.3)	25 (47.2)	0.1284	194.7 (131.5–288.1)	1
Sub-Saharan Africa	249 (12.9)	12 (22.6)	0.0174	687.7 (390.6–1210.9)	3.53 (1.78–7.03)
Other	363 (18.8)	15 (28.3)	0.0233	644.3 (388.4–1068.7)	3.31 (1.75–6.29)
Died during follow-up	226	16			
Age at HIV diagnosis, years, median (range)	33.9 (0–75)	35.4 (6–61)			
Age at start of follow-up, years, median (range)	35.9 (6–75)	35.7 (6–61)			

^a^Time at risk in years per 100,000 persons

Baseline data of all patients in the Helsinki HIV cohort between 1998 and 2015 compared to those in the cohort who developed TB co-infection during follow up. Incidence rates and rate ratios of different subgroups were calculeted with the Mentel-Haenszel method

### Incidence rates

The incidence rates of TB in different subgroups of HIV-positives are shown in Table 1. The incidence rates in the range of 100–700 cases per 100,000 population are very high compared to the current general TB incidence rate of 5 cases per 100,000 in Finland. The TB incidence rate was higher in injecting drug users (IDU) and heterosexuals compared to men having sex with men (MSM). The incidence was lower in those born in Finland compared to migrants from Sub-Saharan Africa and other countries.

To analyze for temporal changes the TB incidence rates were analyzed in 5-year periods (Table 2). This showed a statistically significant reduction in incidences with a 59% decrease per 5-year period, as the incidence rate dropped from 691 to 130 per 100,000. The incidence rate declined significantly in all subgroups, except for in females and in those with country of origin other than Finland or Sub-Saharan Africa. In IDUs, almost all of whom were men from Finland, there was a remarkable reduction in in the incidence rate from 1182 to 88 per 100,000 (*p* = 0.0009, score test for trend of rates).

**Table 2:** Incidence rates of TB divided into three 6-year periods

	Events	Person years^a^	Incidence rate (95% CI)	Score test for trend of rates	*P*-value
				Rate ratio (95% CI)	
Total				0.41 (0.29–0.59)	< 0.001
1998–2003	22	0.0318	691.2 (455.1–1049.7)		
2004–2009	20	0.0543	368.5 (237.7–571.1)		
2010–2015	11	0.0847	129.9 (71.9–234.5)		
Sex					
Female				0.57 (0.31–1.04)	0.068
1998–2003	6	0.0086	698.7 (313.9–1555.3)		
2004–2009	6	0.0144	416.9 (187.3–928.0)		
2010–2015	6	0.0241	248.8 (111.8–553.9)		
Male				0.35 (0.23–0.54)	< 0.001
1998–2003	16	0.0232	688.4 (421.8–1123.7)		
2004–2009	14	0.0399	351.0 (207.9–592.6)		
2010–2015	5	0.0606	82.5 (34.4–198.3)		
Mode of transmission					
IDU				0.29 (0.14–0.60)	< 0.001
1998–2003	8	0.0068	1182.2 (591.2–2363.9)		
2004–2009	3	0.0113	265.0 (85.5–821.6)		
2010–2015	1	0.0114	88.0 (12.4–625.0)		
MSM				0.19 (0.08–0.45)	< 0.001
1998–2003	6	0.0132	454.3 (204.1–1011.2)		
2004–2009	3	0.0220	136.4 (44.0–423.0)		
2010–2015	0	0.0345	0		
Heterosex				0.35 (0.23–0.54)	< 0.001
1998–2003	8	0.0113	709.5 (354.8–1418.6)		
2004–2009	13	0.0196	663.0 (385.0–1141.8)		
2010–2015	9	0.0362	248.3 (129.2–477.3)		
Place of birth					
Finland				0.30 (0.18–0.49)	< 0.001
1998–2003	15	0.0264	567.2 (342.0–940.9)		
2004–2009	7	0.0427	163.9 (78.1–343.7)		
2010–2015	3	0.0593	50.6 (16.3–156.9)		
Sub-Saharan Africa				0.27 (0.12–0.59)	< 0.001
1998–2003	5	0.0025	2017.2 (839.6–4846.4)		
2004–2009	5	0.0047	1063.1 (442.5–2554.1)		
2010–2015	2	0.0103	194.8 (48.7–778.9)		
Other				0.50 (0.24–1.05)	0.066
1998–2003	2	0.0024	829.0 (207.3–3314.7)		
2004–2009	8	0.0063	1261.5 (630.9–2522.5)		
2010–2015	5	0.0145	344.2 (143.3–826.9)		

^a^Time at risk in years per 100,000 persons

Here the incidence rates of TB cases in the HIV cohort are shown divided into three 6-year periods. Score test for trend of rates shows the change in incidence between the 6-year periods. Rate ratios and 95% confidence intervals are calculated with the Mantel-Haenszel method

### Genotyping of M. Tuberculosis isolates

*M. tuberculosis* isolates were available for 38 of the 53 active TB cases, and were successfully genotyped (Table 3). The most common spoligotypes were SIT53 and SIT42, with 7 and 5 cases each. Both of those included small clusters among IDUs born in Finland with active TB infection between years 2000–2003. Based on genotypes, most of the other TB infections were sporadic cases of activation of latent TB infection obtained in the country of origin. There were no signs that *M. tuberculosis* strains from Russia or Estonia would have caused transmission chains among HIV-positives in Finland.

**Table 3:** *M. tuberculosis* genotypes

Spoligotype	Octal Code	Frequency	%
SIT53	777777777760771	7	13.2
SIT42	777777607760771	5	9.4
SIT1	000000000003771	3	5.6
SIT89	674000003413771	3	5.6
SIT149	777000377760771	1	1.9
SIT262	774777777420771	1	1.9
F286	773637777413771	1	1.9
F351	767777777740771	1	1.9
F373	777777657413771	1	1.9
SIT102	777703777760771	1	1.9
SIT106	776177400000171	1	1.9
SIT144	770000003760771	1	1.9
SIT1815	677777606760771	1	1.9
SIT2023	777737777720171	1	1.9
SIT2088	777777737760731	1	1.9
SIT2493	777773606060731	1	1.9
SIT256	777777777413671	1	1.9
SIT2724	774737777420771	1	1.9
SIT345	777000377760731	1	1.9
SIT49	777777777720731	1	1.9
SIT50	777777777720771	1	1.9
SIT75	777767777720771	1	1.9
SIT932	777777663760731	1	1.9
SIT937	777777777013771	1	1.9
NA	NA	15	28.3
Total		53	100

Distribution of *M. tuberculosis* genotypes in HIV patients with active TB infection. *NA* not available

### Outcomes

The 53 HIV-TB co-infected patients were classified (Table 4) according to WHO’s definition of tuberculosis treatment outcomes [1]. Three of the patients died during the TB treatment and 13 after the treatment. Classified as treatment completed or cured were 66.0% of the cases. In 22.6% of the cases data of outcome was not available due to transfer out or other reasons. The average length of TB treatment was 11.7 months.

**Table 4:** Outcomes of TB treatment

Treatment outcomes	n	%
Treatment completed/Cured	35	66.0
Treatment failure	0	0
Died during treatment	3	5.7
Default	3	5.7
Transfer out	5	9.4
Data not available	7	13.2
Total	53	100

The outcomes of the TB treatment of the HIV-TB co-infected patients in our cohort presented according to WHO’s definitions

### Mortality and causes of death

In the Helsinki HIV cohort, 16 (30.2%) TB co-infected patients died during an average follow-up time of 8.8 years (Table 5). One female and 15 males died during follow-up and this difference was statistically significant (p 0.031, Log-rank test). There was a non-significant trend towards higher risk of death for cases born in Finland and in those who were diagnosed with TB more than 6 months after the moment of the HIV diagnosis.

**Table 5:** Survival of HIV-TB co-infected divided into different risk categories

	All	Died	Alive	Hazard ratio (95% CI)	*P*-value
	n	n	n		
Total	53	16	37		
Sex					0.031
Male	36	15	21	1	
Female	17	1	16	0.15 (0.019–1.10)	
Mode of HIV-transmission					0.58
IDU	13	7	6	1.71 (0.53–5.52)	
MSM	9	4	5	1.85 (0.49–7.02)	
Heterosex	29	5	24	1	
Country of birth					0.14
Finland	26	14	12	4.65 (0.61–35.5)	
Sub-Saharan Africa	11	1	10	1	
Other	15	1	14	1.3 (0.081–21.5)	
Time of TB diagnosis					0.066
> 6 months after HIV diagnosis	32	13	19	3.07 (0.87–10.8)	
< 6 months from HIV diagnosis	29	3	26	1	
MDR TB	2	0	2		
MAC co-infection	5	1	4		
TB recurrence	1	0	1		
Age at TB diagnosis, years, mean (range)	38.9 (20–61)	42.9 (26–61)	37.2 (20–56)	1.06 (1.00–1.13)	0.036
CD4 at TB diagnosis, mean (range)	203 (5–859)	258 (5–854)	181 (9–859)	1.00 (0.99–1.00)	0.27
CD4 nadir, mean (range)	131 (1–471)	118 (1–471)	137 (9–458)	1.00 (0.99–1.00)	0.90

Characteristics of HIV-TB co-infected patients grouped base on whether they have died or are alive at the end of the follow-up period. Log-rank test was used to calculate p-values for categorical variables and Wald test (Cox model) for continuous variables. Hazard ratios and their 95% confidence intervals were calculated using Cox model. Hazard ratios for age and CD4 at time of TB diagnosis are per unit increase

Five of the HIV patients had both *M. tuberculosis* and nontuberculous mycobacterium (NTM) infection at the same time. Three of those were *M. avium*, one *M. abscessus*, and one *M. malmoense*. Two of the patients had *M. avium* cultured from the blood. *M. malmoense* was detected from sputum culture. *M. abscessus* was detected by culture from a lymph node biopsy sample. One of the patients with *M. avium* in sputum culture samples died during follow-up, the other four with NTM were alive at the end of follow-up.

To explore the reasons for death of the 16 HIV-TB co-infected patients, we analysed the ICD-10 diagnoses of the underlying cause of death from Statistics Finland. None of the deaths were directly related to the previous TB infection. Five patients had HIV as primary cause of death, with the direct cause of death being *M. avium* infection, pulmonary hypertension, pneumonia, and non-Hodgkin lymphoma for two patients. Seven patients had deaths caused by intoxication or suicide. One died of coronary disease, one of alcoholic liver cirrhosis, one of sub-arachnoid bleeding, and in one case the cause of death remained unknown. Thirteen of the 22 Finnish males with HIV-TB co-infection died during follow-up (59%). Six of these deaths were intoxications or suicides (46%).

The causes of deaths were also classified using the algorithms developed by the Coding of Death in HIV (CoDe) Project related to the EuroSIDA study [16]. Based on these algorithms, 4–6 of the 16 deaths were classified as AIDS-related, as compared to the 5 deaths with HIV as primary cause of death according to the ICD-10 diagnose in the death certificate.

## Discussion

During the last 20 years, the HIV incidence in Finland has been slightly increasing, mainly because of immigration from Africa and Asia. The tuberculosis incidence has been gradually decreasing, as the number of elderly people with exposure in childhood has declined.

The decline in the incidence of tuberculosis among HIV-infected shown in this study was expected. A comprehensive care center for marginalized HIV-infected IDUs was started in December 2000 [17]. The outbreak of HIV among IDUs in the Helsinki region was contained in a couple of years after the start of the care center [18]. A symptom-based interview of the clients of the care center was carried out in 2002 after the fourth case of tuberculosis among IDUs was identified. The interview yielded one additional case of tuberculosis.

We do not have a definite explanation for the decline in tuberculosis incidence among people from Sub-Saharan Africa. However, the increase in antiretroviral therapy coverage may have played a central role. The coverage of antiretroviral therapy among people from Sub-Saharan Africa in our clinical database has been rising continuously, in 1998 63%, in 2005 65%, in 2010 74%, and in 2015 93% (unpublished data).

The genotypes of the *Mycobacterium tuberculosis* strains showed that imported TB cases have not caused chains of infections among HIV-patients in Finland. Most TB infections in migrants have been activations of latent tuberculosis infections received in the country of birth. Only two small clusters (SIT53 and SIT42) among Finnish IDUs in the beginning of the 2000s could be recognized. This suggests that TB infections in HIV-positives in Finland are mostly diagnosed and treated at an early stage and do not cause a significant public health concern.

The rate of successful treatment of TB was 66% in our Finnish cohort. This can be compared to WHO’s global numbers of treatment success rate of 88% for HIV-negative and 74% for HIV-positive TB-patients [1]. In Europe, the success rate for all TB treatments has increased from 67 to 75% between 1995 and 2012. For new HIV-positive TB-cases registered in Europe in 2013 the TB treatment success rate was 47% in a cohort of 9504 patients. In both the European region and the Region of the Americas, 19% of HIV-positive TB patients died during treatment, compared with just over 5% of HIV-negative TB patients.

A study from Uganda with 302 HIV-TB co-infected enrolled 2007–2009 reported an all cause mortality of 20% during an average follow up time of about one year [6]. Among our 11 HIV-TB coinfected patients from Sub-Saharan Africa only one died, despite longer follow up times. This could be explained by early initiation of ART, which was shown to be the strongest factor protecting from death in the study from Uganda.

There was a rather high number of loss to follow-up seen in the HIV-TB patients in this cohort. Probable reasons were that a substantial part of the subjects were migrants living only temporary in Helsinki. Also IDUs might have a more unregular living situation compared to other residents.

The average duration of TB treatment was nearly 1 year. This cannot be explained by drug resistance, as only two cases of MDR TB were diagnosed. Instead, this seems to reflect the fact that most of the TB cases were treated before year 2006, when the first national Finnish guideline for TB treatment was published, which recommended shorter treatments than earlier. Until that there had been a tradition of long treatment durations in Finland.

The small number of deaths in each subgroup has to be considered when interpreting factors affecting mortality. The all-cause mortality of HIV-TB co-infections in our cohort during an average follow up time of 9 years is 30%. However, none of these 16 deaths were directly related to TB. Among the 16 fatalities, 14 were Finnish males. The all-cause mortality of Finnish males with HIV-TB co-infection was 59%. The reason for this high mortality rate is most likely an accumulation of other risk factors of early death, such as heavy alcohol consumption, smoking, injecting drug use and psychiatric illness. To reduce the mortality rate in this group, active follow-up and multidisciplinary social and psychiatric support could be useful. On the other hand, among HIV-positive females and migrants, TB-infection does not seem to affect their long-term survival.

The benefits of isoniazid preventive therapy (IPT) for latent tuberculosis has remained controversial as most of the randomized trials showing efficacy of this strategy where conducted in Africa before the beginning of highly active antiretroviral therapies [19–21]. The recent TEMPRANO study from Ivory Coast showed that 6 months of IPT has a durable protective effect in reducing mortality in HIV-infected, even in people with high CD4 cell counts and who have started ART [22, 23]. HIV cohort studies from Switzerland and the UK have shown a significant reduction in incidence of active TB after treatment of latent TB [4, 5]. However, the number needed to treat to avoid one case of active TB was 15 and among those with ART as high as 35 [4]. During the time period of our study, IPT was not routinely used to prevent TB in our center.

## Conclusions

To reduce the negative health effects of HIV-TB co-infections in Finland and similar high-income countries, the most important means are early diagnosis and treatment of both HIV and tuberculosis. It is important to identify those HIV-patients with high TB-risk to be able to detect active TB infections as early as possible based on symptoms, chest x-ray, sputum samples and biopsy of enlarged lymph nodes. The treatment results of TB and the long-term prognosis are generally good. However, for non-migrant males with HIV-TB co-infection, the all-cause mortality is unacceptably high and special efforts are needed to address this problem.

## Abbreviations

HUS: Hospital District of Helsinki and Uusimaa
IDU: Injecting drug user
IGRA: Interferon gamma release assay
IPT: Isoniazid preventive therapy
LTBI: Latent tuberculosis infection
NTM: Nontuberculous mycobacterium
TB: Tuberculosis
TST: Tuberculin skin test
